# Light shed on a non-canonical TCA cycle: cell state regulation beyond mitochondrial energy production

**DOI:** 10.1038/s41392-022-01060-5

**Published:** 2022-06-28

**Authors:** Ivona Mateska, Vasileia Ismini Alexaki

**Affiliations:** Institute of Clinical Chemistry and Laboratory Medicine, University Hospital, Technische Universität Dresden, Dresden, Germany

**Keywords:** Biochemistry, Cell biology

**Fig. 1:**
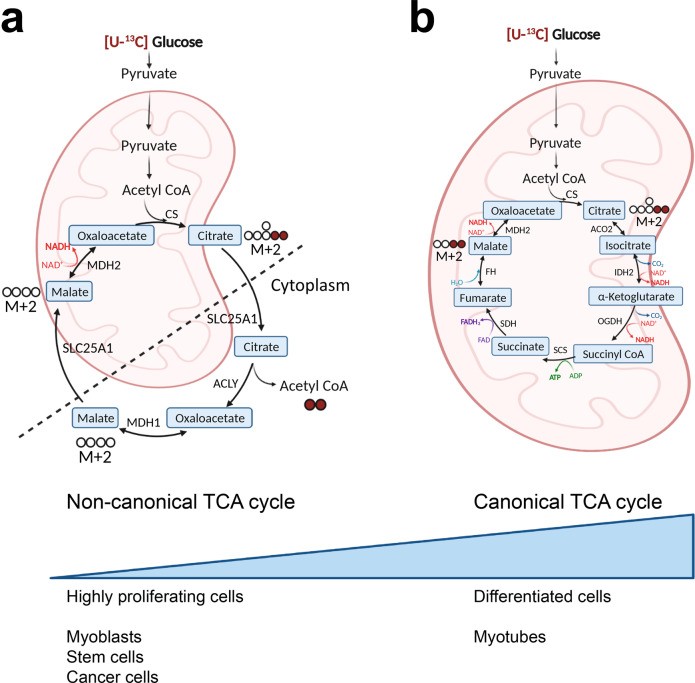
Schematic representation of the two modules of the TCA cycle. **a** non-canonical TCA cycle in proliferating cells, where citrate is exported to the cytoplasm and cleaved by ACLY to produce acetyl-CoA and regenerate oxaloacetate, which yields malate that is transported back to mitochondria. **b** canonical TCA cycle from citrate to malate in mitochondria, mainly present in differentiated cells. Tracing of M+2 citrate derived from [U-^13^C]glucose to M+2 malate, when metabolized by aconitase (ACO) in the canonical TCA cycle (**b**), or to unlabeled malate, when metabolized by ACLY in the cytoplasm (**a**), is shown. Created with BioRender.com

A recent publication in *Nature* by Arnold et al. reports on the discovery of a non-canonical tricarboxylic acid (TCA) cycle in which acetyl-CoA produced from mitochondrially derived citrate by ACLY regenerates oxaloacetate in the cytoplasm.^1^ Oxaloacetate is converted through a two-direction reaction to malate, which is uptaken by the mitochondrial citrate/malate transporter SLC25A1 in the mitochondria, where it fuels oxaloacetate and citrate production (Fig. 1a).

The TCA cycle, also known as Krebs cycle, was discovered eight decades ago by the biochemist Hans Krebs. It is often regarded as a catabolic pathway combusting carbohydrates and lipids to ultimately produce energy in the form of ATP through its coupling to oxidative phosphorylation.^2^ It consists of a loop of reactions known to take place in mitochondria and fueled by acetyl-CoA, which is generated from pyruvate or fatty acids and oxidized into CO_2_. Acetyl-CoA reacts with oxaloacetate generating citrate, which is then transformed to its isomer isocitrate. The latter is converted to α-ketoglutarate, which is metabolized to succinyl-CoA releasing two CO_2_ and NADH molecules. Succinyl-CoA is further converted to succinate along with GTP generation. Oxidation of succinate by succinate dehydrogenase, which is Complex II of the electron transfer chain, generates fumarate and two FADH_2_ molecules. Fumarate is metabolized to malate and then to oxaloacetate, which is used for citrate production closing the loop of reactions. NADH and FADH_2_ formed in the TCA cycle deliver electrons to the electron transfer chain, thereby fueling ATP generation^2^ (Fig. 1b). Besides energy production, the TCA cycle also serves anabolic reactions through the production of metabolites, which can be shuttled from the mitochondria to the cytoplasm. Importantly, citrate can be transported through SLC25A1 outside of the mitochondria and transformed to acetyl-CoA and oxaloacetate by ATP citrate lyase (ACLY). Acetyl-CoA serves as the building block for fatty acid synthesis, while it also provides acetyl groups for posttranslational protein modifications, including histone acetylation, thereby relaying the TCA cycle to epigenetic regulation.^2^

In this recent study,^1^ the authors analyzed the metabolic gene essentiality scores from genome-wide loss of function CRISPR screens in 769 human cancer cell lines and noticed that TCA cycle-associated genes clustered in two separate groups: one forming the traditional TCA cycle and another related to a non-canonical TCA cycle module. They monitored both modules with elegant tracing studies using [U-^13^C]glucose which generates citrate labeled with two ^13^C atoms (M+2). Metabolism of the M+2 citrate by mitochondrial aconitase generates M+2-labeled TCA cycle intermediates (Fig. 1b), whereas its engagement in the non-canonical TCA cycle through ACLY releases the ^13^C atoms into acetyl-CoA, generating unlabeled oxaloacetate and downstream metabolites (Fig. 1a). Hence, M+2 labeling in this set of experiments reflected the degree to which cells commit to the non-canonical versus the canonical TCA cycle. In the non-small cell lung cancer cell lines studied, ACLY inhibition increased the M+2-labeled malate / M+2-labeled citrate ratio. Similarly, in non-transformed embryonic stem (ES) cells disruption of ACLY or aconitase lead to increase or decrease in the commitment to the canonical TCA pathway, respectively, indicating that ES cells are relatively flexible to switch between the two TCA cycle modes. Moreover, these manipulations highlighted the important role of ACLY in determining the intracellular levels of citrate, fumarate and malate. Taking this a step further, deuterium tracking from [4-^2^H]glucose through NADH to malate and citrate, provided proof that cytosolic malate contributes to citrate regeneration in mitochondria in an ACLY-dependent manner, thereby closing the loop of reactions of the non-canonical TCA cycle. As a result, ACLY inhibition negatively affected mitochondrial oxygen consumption. SLC25A1 and malate dehydrogenase 1 (MDH1) disruption mimicked ACLY inhibition, thereby establishing that the axis SLC25A1-ACLY-MDH1 forms the non-canonical TCA cycle in ES cells. To reconcile the new findings with the established knowledge on the mitochondrial TCA cycle, authors traced [U-^13^C]glucose derivatives in cultured myoblasts and differentiated myotubes, given that the original experiments by Krebs were performed in pigeon breast muscle.^3^ Interestingly, undifferentiated myoblasts showed a higher degree of non-canonical TCA cycle activity compared to differentiated myotubes, which switched to the traditional TCA cycle, suggesting that the cell differentiation state determines which TCA cycle module is mainly used. The preference of myoblasts for the non-canonical and of myotubes for the canonical pathway was also reflected at the TCA cycle gene expression level, indicating that TCA cycle rewiring may be regulated at the transcriptional level. Importantly, ES cells were dependent on citrate metabolism through cytoplasmic ACLY and required an ACLY-dependent switch from the canonical to the non-canonical TCA cycle to exit from the naïve pluripotent state and maintain their viability and proliferation.

Although the exact mechanisms underlying the regulation of cell identity by the non-canonical TCA cycle were not delineated, gene regulation through acetyl-CoA-dependent histone acetylation would be a plausible mechanism to study in this context. Epigenetic histone modifications requiring TCA cycle metabolites has opened the question of the necessity for timely and dynamic nuclear availability of these metabolites. Along this line, TCA cycle enzymes, such as pyruvate dehydrogenase, were found to be transiently located and active in the nucleus of cells of the early stage embryo where they regulate epigenetic remodeling required for zygotic genome activation.^4^

In conclusion, the here highlighted findings emphasize the dynamic and plastic nature of the TCA cycle in response to cell state-specific energetic or anabolic demands, allowing for cell identity transitions. The ACLY-driven non-canonical TCA cycle is suggested to repurpose the function of the TCA cycle from carbohydrate and lipid consumption to retaining reduced carbon metabolites, regeneration of NAD^+^ required to sustain glycolysis and production of acetyl-CoA for building up lipids and promoting histone acetylation. These metabolic traits may be critical for embryonic stem cells, cancer cells and inflammatory immune cells, which are all cell states with high anabolic demands due to their accelerated proliferation and protein and lipid production. Hence, a deeper understanding of the role of the non-canonical TCA cycle in cancer and immune cells may offer valuable clues on the manipulation of their function in disease.
